# Polymorphisms in the *ASMT* and *ADAMTS1* gene may increase litter size in goats

**DOI:** 10.1002/vms3.301

**Published:** 2020-06-11

**Authors:** Wenping Hu, Jishun Tang, Zhuangbiao Zhang, Qianqian Tang, Yan Yan, Pinqing Wang, Xiangyu Wang, Qiuyue Liu, Xiaofei Guo, Mei Jin, Yingjie Zhang, Ran Di, Mingxing Chu

**Affiliations:** ^1^ Key Laboratory of Animal Genetics and Breeding and Reproduction of Ministry of Agriculture and Rural Affairs Institute of Animal Sciences Chinese Academy of Agricultural Sciences Beijing PR China; ^2^ Institute of Animal Husbandry and Veterinary Medicine Anhui Academy of Agricultural Sciences Hefei PR China; ^3^ Bioengineering College Chongqing University Chongqing PR China; ^4^ College of Life Science Liaoning Normal University Dalian PR China; ^5^ College of Animal Science and Technology Agricultural University of Hebei Baoding PR China

**Keywords:** *ADAMTS1* gene, *ASMT* gene, goat, reproduction, single nucleotide polymorphisms

## Abstract

Prolificacy of most local goat breeds in China is low. Jining Grey goat is one of the most prolific goat breeds in China, it is an important goat breed for the rural economy. ASMT (acetylserotonin O‐methyltransferase) and ADAMTS1 (ADAM metallopeptidase with thrombospondin type 1 motif) are essential for animal reproduction. Single nucleotide polymorphisms (SNPs) of *ASMT* and *ADAMTS1* genes in the highly prolific breed (Jining Grey goats), medium prolific breed (Boer goats and Guizhou White goats) and low prolific breeds (Angora goats, Liaoning Cashmere goats and Inner Mongolia Cashmere goats) were detected by polymerase chain reaction‐restriction fragment length polymorphism and sequencing. Two SNPs (g.158122T>C, g.158700G>A) of *ASMT* gene and two SNPs (g.7979798A>G, g.7979477C>T) of *ADAMTS1* gene were identified. For g.158122T>C of *ASMT* gene, further analysis revealed that genotype TC or CC had 0.66 (*p* < 0.05) or 0.75 (*p* < 0.05) kids more than those with genotype TT in Jining Grey goats. No significant difference (*p* > 0.05) was found in litter size between TC and CC genotypes. The SNP (g.158122T>C) caused a p.Tyr298His change and this SNP mutation resulted in changes in protein binding sites and macromolecule‐binding sites. The improvement in reproductive performance may be due to changes in the structure of ASMT protein. For g.7979477C>T of *ADAMTS1* gene, Jining Grey does with genotype CT or TT had 0.82 (*p* < 0.05) or 0.86 (*p* < 0.05) more kids than those with genotype CC. No significant difference (*p* > 0.05) was found in litter size between CT or TT genotypes. These results preliminarily indicated that C allele (g.158122T>C) of *ASMT* gene and T allele (g.7979477C>T) of *ADAMTS1* gene are potential molecular markers which could improve litter size of Jining Grey goats and be used in goat breeding.

## Graphical abstract

ASMT (acetylserotonin O‐methyltransferase) and ADAMTS1 (ADAM metallopeptidase with thrombospondin type 1 motif) are very important for female and animal reproduction. One mutation (g.158122T>C) of *ASMT* gene and one mutation (g.7979477C>T) of *ADAMTS1* gene were found in goats in this study, and the association between polymorphisms of *ASMT* or *ADAMTS1* genes and prolificacy in Jining Grey goats were investigated. It suggested the *ASMT* gene with C allele and *ADAMTS1* gene with T allele may increase the litter size in goats.

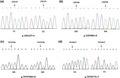

## 1 INTRODUCTION

Acetylserotonin O‐methyltransferase (ASMT) is the last enzyme of the melatonin (MLT) synthesis pathway (Botros et al., 2013). The biosynthesis of MLT from serotonin needs two enzymatic steps. First, serotonin N‐acetyltransferase catalyses serotonin to N‐acetylserotonin (NAS), and then ASMT catalyses NAS to O‐methylated NAS, also known as MLT (Byeon & Back, 2016).

Melatonin is an important hormone synthesized and secreted by the pineal gland and retina in dark. It plays important roles in physiological functions including regulating the biological clock, circadian rhythms, animal reproduction and other activities (Dollins, Zhdanova, Wurtman, Lynch, & Deng, 1994). One of the important roles of MLT is to regulate the reproductive system, which can directly act on the gonads to control the secretion of reproductive hormones and also can indirectly influence the gonad to regulate the secretion of reproductive hormones through acting on the anterior pituitary firstly (Recio, Mediavilla, Cardinali, & Sanchez‐Barcelo, 1994). Animals with seasonal reproductive characteristics can be divided into short‐day and long‐day reproductive animals. The role of MLT on the two kinds of animals is the opposite, which can inhibit sexual activities of the long‐day animals but stimulate the short‐day ones.

Up to now, *ASMT* gene of goat had been cloned and sequenced, which is 28,511 bp (*Capra hircus*, NW_017189541.1). *ASMT* gene was expressed in bovine cumulus oocyte complexes (COCs). Melatonin receptors presented in COCs, and MLT could significantly enhance oocyte nuclear maturation and cumulus cell expansion, it indicated the potentially important roles of MLT in regulating bovine oocyte maturation (El‐Raey et al., 2011). ASMT is important for animal reproduction. It was expressed in human placenta throughout pregnancy (Soliman et al., 2015). There is however paucity of information on *ASMT* gene in goats.

ADAMTS1 (ADAM metallopeptidase with thrombospondin type 1 motif) protein is a multidomain, multifunctional metalloprotease. ADAMTS belongs to matrix metalloproteinase family. In the amino‐terminal region of ADAMTS1, it contains metalloproteinase and disintegrin‐like domains (Willis, Bridges, & Fortune, 2017). As a multifunctional protease, ADAMTS1 is capable of cleaving matrix proteoglycans such as aggrecan, versican and brevican (Kuno et al., 2000; Rodriguez‐Manzaneque et al., 2002; Sandy et al., 2001; Yuan, Matthews, Sandy, & Gottschall, 2002).

The length of *ADAMTS1* gene in goat is 9,470 bp (*Capra hircus*, NC_030808.1). ADAMTS plays essential roles in various diseases as well as organogenesis (Hirohata, Inagaki, & Ohtsuki, 2017; Kunkle et al., 2019; Tan, Frewin, Ricciardelli, & Russell, 2019), and is likely to be necessary for organ morphology and function, normal growth, and fertility. ADAMTS1 is very important for female reproduction. The newest clinical research suggested that ADAMTS1 was involved in the pathogenesis of polycystic ovary syndrome (Karakose et al., 2016; Tola, Karatopuk, Koroglu, Ergin, & Oral, 2017). Insufficiency of ADAMTS1 expression in Sertoli cells may be related to male infertility, and it could be a potential diagnostic marker in male azoospermia (Aydos, Yukselten, Ozkavukcu, Sunguroglu, & Aydos, 2019).

In the granulosa cells of the preovulatory follicles, *ADAMTS1* mRNA expression could be induced by administering luteinizing hormone (LH), and the expression level was sustained in a progesterone‐dependent manner (Boerboom, Russell, Richards, & Sirois, 2003; Espey et al., 2000; Robker et al., 2000; Sayasith, Lussier, & Sirois, 2013). *ADAMTS1* was one of the extracellular signal‐regulated kinase 1/2 (ERK1/2) dependent LH‐induced genes (Schuermann et al., 2018) and upregulated in bovine granulosa cells during ovulation (Lussier, Diouf, Levesque, Sirois, & Ndiaye, 2017). The expression of *ADAMTS1* gene in ovaries of biparous Mongolian sheep was about 30 fold higher than that of monotocous Mongolian sheep (He et al., 2012). *ADAMTS1* mRNA was expressed in endometria, conceptus, and placentomes (Dunlap et al., 2010).

Both ASMT and ADAMTS1 are important for animal reproduction (Brown & Russell, 2014; El‐Raey et al., 2011; Mishra et al., 2013). Literature on polymorphisms of caprine *ASMT* and *ADAMTS1* genes and their association with reproductive traits is rare. Goats reared in P.R. China display different litter sizes, which provide the materials to analyse the association of *ASMT* and *ADAMTS1* genes with prolificacy in goats. Most of the local goat breeds are low prolific in China, Jining Grey goat is one of the most prolific caprine breeds in P.R. China, and Jining Grey goat displays significant characteristics of year‐round oestrus, and sexual precocity, it is an important goat breed for the rural economy. Sexual maturity of Jining Grey goats is at 3–4 months, the first mating age of that is 5–7 months. Sexual maturity of Guizhou White goats and Boer goats is 4–6 months, the first mating age of that is 6–8 months. Sexual maturity and the first mating age of Angora goats, Liaoning Cashmere goats, and Inner Mongolia Cashmere goats are the latest, about 6–8 months and 18 months respectively. The meanlitter sizes of Jining Grey goats, Guizhou White goats, Boer goats, Angora goats, Liaoning Cashmere goats, and Inner Mongolia Cashmere goats were reported to be 2.83, 2.13, 2.10, 1.31, 1.15 and 1.05 respectively (Malan, 2000; Roberts & Reeves, 1988; Tu, 1989). Single nucleotide polymorphisms (SNPs) of *ASMT* and *ADAMTS1* genes were identified and the association of polymorphisms with litter size in goat were investigated in this study. So as to acquire molecular markers related to prolificacy for marker‐assisted selection.

## 2 MATERIALS AND METHODS

### 2.1 Genomic DNA isolation

#### 2.1.1 Genomic DNA isolation for *AMST* gene

Venous jugular blood samples (10 ml per goat doe) were collected from 296 Jining Grey does (Jining Grey Goats Conservation Base, Jiaxiang County, Shandong Province, PR China), 60 Boer and 60 Angora does (Qinshui Demonstration Farm, Qinshui County, Shanxi Province, PR China), 60 Liaoning Cashmere does (Liaoning Cashmere Goat Breeding Center, Liaoyang City, Liaoning Province, PR China), 44 Inner Mongolia Cashmere does (Inner Mongolia White Cashmere Goat Breeding Farm, Etuokeqi, Ordos City, the Inner Mongolia Autonomous Region, PR China). Ten milliliter blood per doe was collected with vacutainer from the jugular vein. Genomic DNA was extracted using TIANamp Blood DNA kit (Tiangen Biotech Beijing CO., LTD.) and then dissolved in TE buffer and stored at −20°C.

The 296 Jining Grey does were selected at random and were the progeny of five goat bucks (*n* = 55, 57, 60, 61, 63). No selection on litter size or other fertility traits was conducted in the flock over previous years. Kidding seasons consisted of 3‐month groups starting with March to May as season 1 (spring, *n* = 76), June to August as season 2 (summer, *n* = 68), September to November as season 3 (autumn, *n* = 88) and December to February as season 4 (winter, *n* = 64).

#### 2.1.2 Genomic DNA isolation for *ADAMTS1* gene

Blood samples were collected from 243 Jining Grey does (Jining Grey Goats Conservation Base, Jiaxiang County, Shandong Province, China), 55 Guizhou White does (Guizhou White Goat Breeding Farm, Yanhe Tujia Nationality Autonomous County, Guizhou Province, China), 32 Boer does (Qinshui Demonstration Farm, Qinshui County, Shanxi Province, China), 82 Liaoning Cashmere does (Liaoning Cashmere Goat Breeding Center, Liaoyang City, Liaoning Province, China) and 60 Inner Mongolia Cashmere does (Inner Mongolia White Cashmere Goat Breeding Farm, Etuokeqi, Ordos City, the Inner Mongolia Autonomous Region, China). Genomic DNA isolation method was the same with *AMST* gene.

The 243 Jining Grey does were randomly selected from the progeny of five goat bucks (*n* = 42, 46, 50, 51, 54). There was no selection on litter size or other fertility traits in this population over the years. Kidding happened in year‐round: spring, *n* = 64; summer, *n* = 56; autumn, *n* = 71; and winter, *n* = 52.

### 2.2 PCR amplification

Three pairs of primers (P1–P3) were designed to amplify the exon 8 and 3′ flanking region of goat *ASMT* gene (GenBank No. NW_017189541.1) by Primer Premier 5.0 from 10 does of both Jining Grey and Liaoning Cashmere goats randomly selected respectively (Table 1). Seven pairs of primers (P1–P7) were designed to amplify the exon 5–9 and 3′ flanking region of goat *ADAMTS1* gene (GenBank No. NC_030808.1; Table 2).

**TABLE 1:** Amplified region, product size and annealing temperature for three pairs of primers used to amplify goat *ASMT* gene

Primer	Primer sequence (5′ → 3′)	Amplified region	Product size (bp)	Annealing temperature *T*_m_ (°C)
P1	F: ATCCTGGTCATCGAGAGCCT R: CCTCTTGGACTCTATGGTG	Exon 8 and 3′ flanking region	914	56
P2	F: ATCCTGGTCATCGAGAGCCT R: GCCAAGACTGCATCGTAGGT	Exon 8	194	61
P3	F: AACCCAGCGACAAGGTCCT R: GTTCCCGCCTCTTCCAGCTT	3′ flanking region	153	60

**TABLE 2:** Amplified region, product size and annealing temperature for seven pairs of primers used to amplify goat *ADAMTS1* gene

Primer	Primer sequence (5′ → 3′)	Amplified region	Product size (bp)	Annealing temperature *T*_m_ (°C)
P1	F: GAAGGGCTGATGCACTGAAATC	3′ flanking region	789	60
	R: TCTTACCGACTCTCTTCAGAC			
P2	F: CCTCATACAGCTCCCCTCTGAT	Exon 5	257	60
	R: CAAAGTGTTCCCTGTTGGTCTG			
P3	F: ACCCCTGTTCACGGAAGCTG	Exon 6	187	62.2
	R: CGCTATTCTTCGGGCAGTCCTC			
P4	F: AACCTTTAGGCTGGAACAGTGTG	Exon 7	169	59
	R: GGGCTGCAAAACGAAGAAATAGC			
P5	F: TGGTGGATGGCACCCCATGT	Exon 8	175	58
	R: CTTGCACTGGTAACTGATCCTG			
P6	F: ACCCGGCTACCATGATATCGT	Exon 9	699	58
	R: TAACTGCACTCTGCCGTTGTG			
P7	F: CCTCATACAGCTCCCCTCTGAT	Exon 5	221	60
	R: CGTTGACACACCATTTCCCCTCTGC			

The PCR mixture contained 2 μl of dNTPs (2.5 mM each), 1 μl of genomic DNA (200 ng/μl), 0.2 μl (5 U/μl) of LA Taq DNA polymerase (Takara, Dalian, China), 0.5 μl (20 μM) of each forward and reverse primers, and 12.5 μl of 2× GC buffer in a 25 μl volume, and run on a Mastercycler^®^ 5,333 (Eppendorf AG). The PCR amplification program was: 95°C for 3 min, 30 cycles of 94°C for 40 s, annealing for 1 min (annealing temperature is shown in Table 1), and 72°C for 1 min and a final extension at 72°C for 10 min.

### 2.3 Cloning and sequencing

PCR products were recovered using Geneclean Ⅱ kit (Promega), and then ligated into the pGEM‐T Easy vector (Promega) at 16°C overnight according to the manufacturer's instructions. After ligation, DNA was transformed into the competent cell (*Escherichia coli* DH5α). Positive clones were identified by the restriction enzyme and then sequenced by Sangon Biotech (Shanghai) Co. Ltd.

### 2.4 Restriction fragment length polymorphism analysis

After sequence alignment, polymorphisms of *ASMT* and *ADAMTS1* gene were screened between Liaoning Cashmere goat and Jining Grey goat. Primers P3 and P7 were used for polymerase chain reaction‐restriction fragment length polymorphism (PCR‐RFLP) to detect the polymorphisms for five goat breeds respectively. The mixture for PCR‐RFLP of *ASMT* gene was: 5 U of the restriction enzyme Afa I or Msp I (Takara), 5 μl of PCR products and 1 μl 10× reaction buffer. The mixture for PCR‐RFLP of *ADAMTS1* gene was: 5 U of the restriction enzyme Nco Ⅰ or Hha I (Takara), 5 μl of PCR products, and 1 μl 10× reaction buffer. The mixtures were incubated at 37°C for 4 hr, and then separated on a 12% polyacrylamide gel at 120 V. After electrophoresis, the DNA fragments in the gels were visualized by silver nitrate staining, photographed and analysed using an AlphaImager^™^ 2,200 and 1,220 Documentation and Analysis Systems (Alpha Innotech Corporation).

### 2.5 Statistical analysis

Analysis of litter size in Jining Grey goat was performed using the following fixed effects model. Least squares mean was used for multiple comparisons in litter size among different genotypes.

$$Y_{\mathrm{ijklm}}=m+S_{i}+{\text{KS}}_{j}+P_{k}+G_{l}+e_{\mathrm{ijklm}},$$

where *Y_ijklm_* is the phenotypic value of litter size; *µ* is the population mean; *S_i_* is the fixed effect of the *i*th sire (*i* = 1, 2, 3, 4, 5); KS*_j_* is the fixed effect of the *j*th kidding season (*j* = 1, 2, 3, 4); *P_k_* is the fixed effect of the *k*th parity (*k* = 1, 2, 3); *G_l_* is the fixed effect of the *l*th genotype (*l* = three different genotypes); and *e_ijklm_* is the random residual effect of each observation.

The general linear model and mean separation procedures of SAS (Ver 8.1; SAS Institute Inc.) were used to analyse the least significant differences.

### 2.6 Bioinformatics analysis

First, the integral and coding sequences of gene were obtained from NCBI (https://www.ncbi.nlm.nih.gov/), the amino acid sequences were obtained from UniProt (https://www.uniprot.org/). Prediction of the secondary structure of gene and its mutants was carried out using PredictProtein (https://www.predictprotein.org/). The 3‐dimension structure before and after mutation in gene were predicted via Phyre2 (http://www.sbg.bio.ic.ac.uk/phyre2/html/page.cgi?id=index).

## 3 RESULTS

### 3.1 PCR amplicons of goat *ASMT* and *ADAMTS1* genes

**FIGURE 1:**
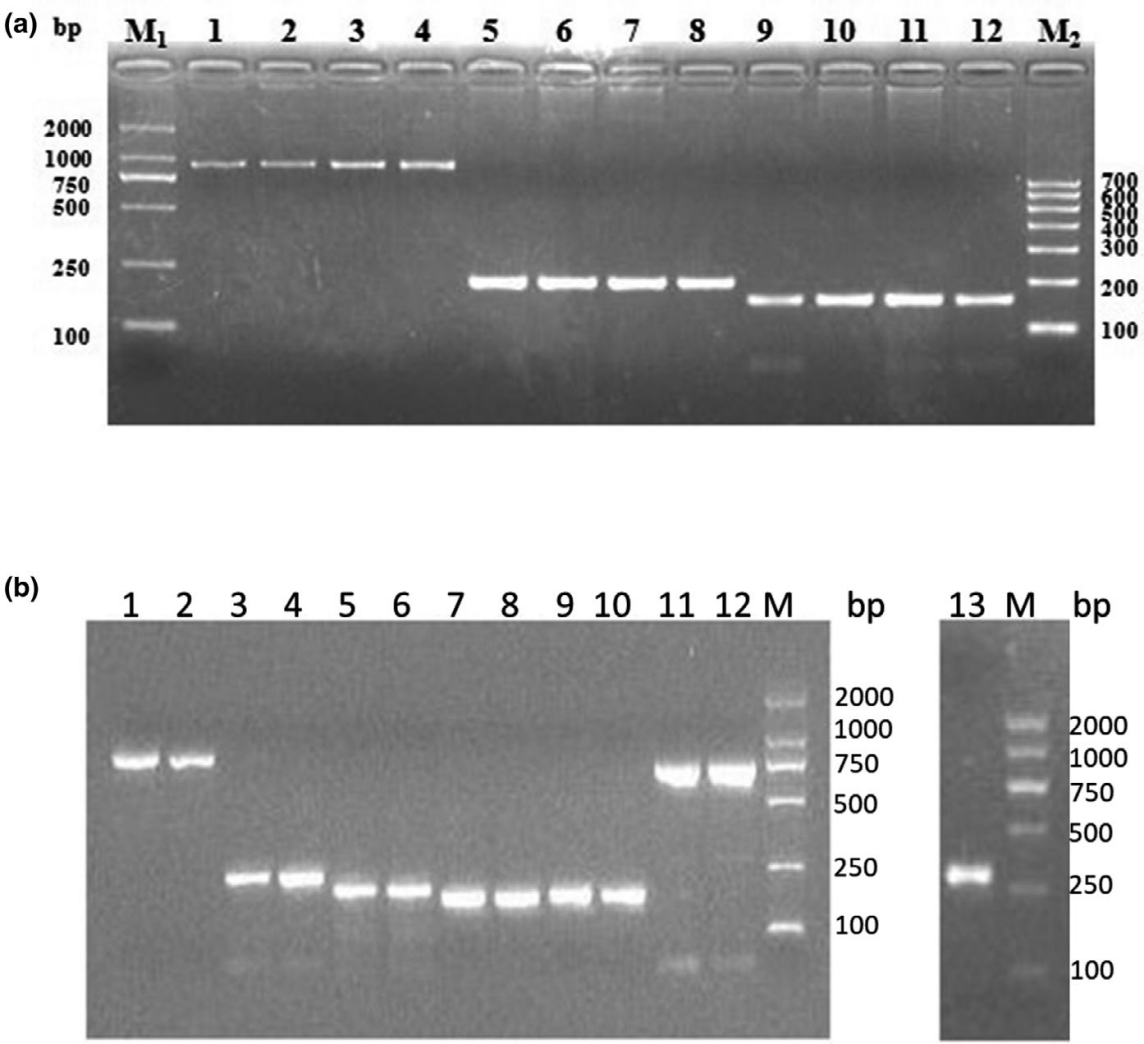
PCR products of *ASMT* and *ADAMTS1* genes. (a) PCR products of the *ASMT* gene. M1: DL 2,000 DNA Marker (Takara, Dalian); Lanes 1–4: primer P1; Lanes 5–8: primer P2; Lanes 9–12: primer P3; M2: DL 700 DNA Marker (Biomed, Beijing). (b) PCR products of *ADAMTS1* gene. M: DL 2,000 DNA Marker (Takara, Dalian); Lanes 1 and 2: primer P1; Lanes 3 and 4: primer P2; Lanes 5 and 6: primer P3; Lanes 7 and 8: primer P4; Lanes 9 and 10: primer P5; Lanes 11 and 12: primer P6; Lane 13: primer P7

Using PCR with the primers P1–P3, the *ASMT* gene was successfully amplified (Figure 1a). *ADAMTS1* gene was also successfully amplified by primers P1–P7 (Figure 1b). The 2% agarose gels were used to separate the PCR products. It showed that the sizes of amplified fragments and the target ones were consistent and the specificity of amplification results was apparent. It could be directly analysed by RFLP and sequencing.

### 3.2 RFLP for *ASMT* and *ADAMTS1* genes

**FIGURE 2:**
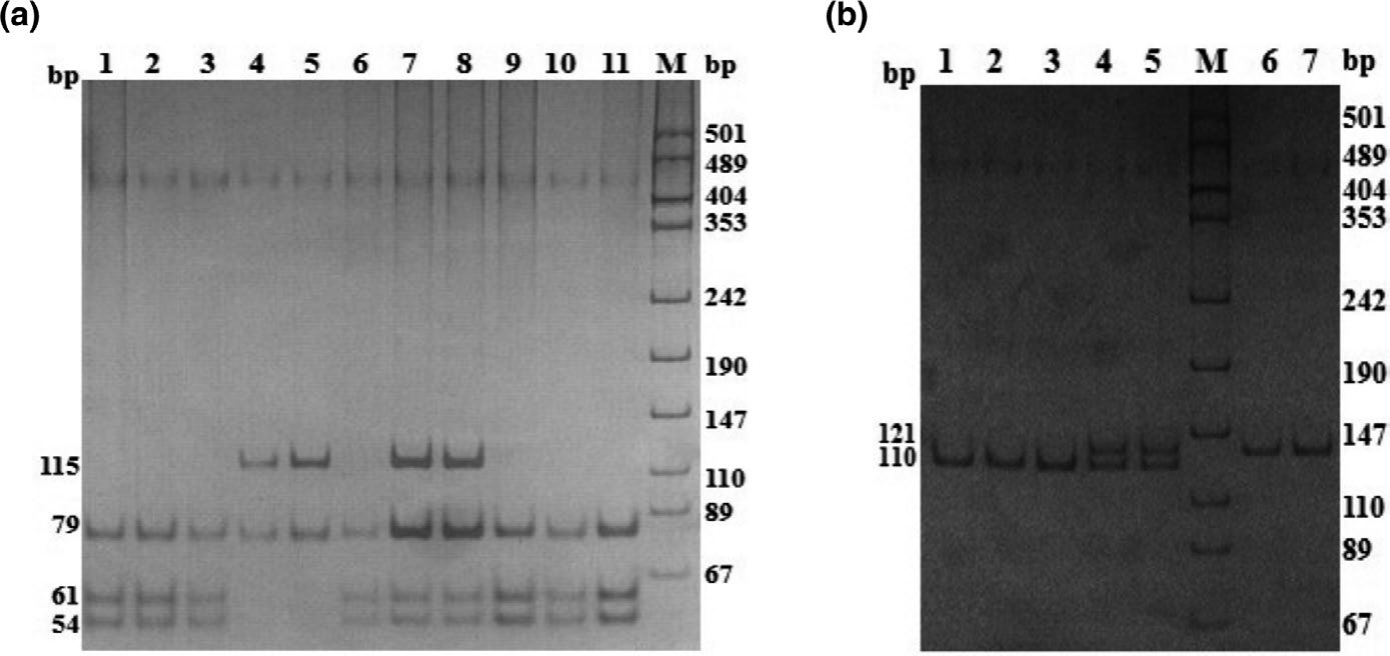
RFLP analysis of PCR products of the *ASMT* gene (12% neutral polyacrylamide gel stained with silver nitrate). (a) RFLP (Afa I) analysis of PCR products of primer P2. Lanes 1, 2, 3, 6, 9, 10 and 11: TT genotype; Lanes 4 and 5: CC genotype; Lanes 7 and 8: TC genotype; M: pUC18 DNA/MspⅠ (Tiangen, Beijing). (b) RFLP (Msp I) analysis of PCR products of primer P3. Lanes 1–3: GG genotype; Lanes 4 and 5: GA genotype; Lanes 6 and 7: AA genotype; M: pUC18 DNA/MspⅠ (Tiangen, Beijing)

The PCR products of *ASMT* gene amplified by primer P2 were digested by Afa I and displayed three genotypes: TT (79/61/54 bp), TC (115/79/61/54 bp) and CC (115/79 bp; Figure 2a). The PCR products of *ASMT* gene amplified by P3 were digested by Msp I and displayed three genotypes: GG (122/22/9 bp), GA (131/122/22/9 bp) and AA (131/22 bp; Figure 2b).

**FIGURE 3:**
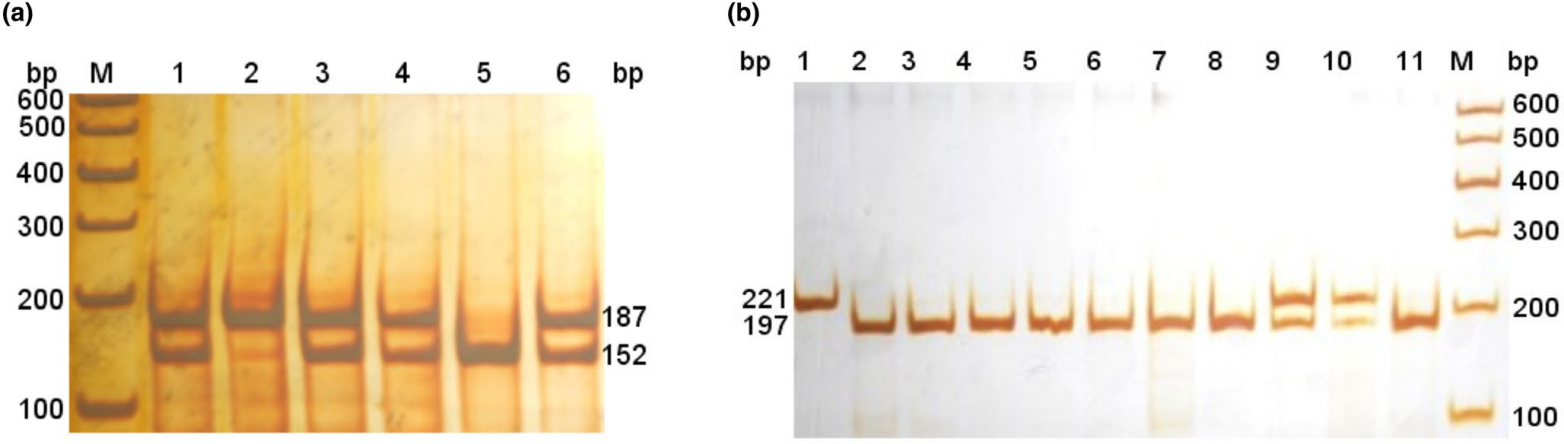
RFLP analysis of PCR products of *ADAMTS1* gene. (a) The result of primer P3, M: DNA MarkerⅠ (Biomed, Beijing); Lane 5: AA genotype; Lanes 1, 3, 4 and 6: AG genotype; Lane 2: GG genotype. (b) The result of primer P7, M: DNA MarkerⅠ (Biomed, Beijing); Lane 1: TT genotype; Lanes 9 and 10: CT genotype; Lanes 2, 3, 4, 5, 6, 7, 8 and 11: CC genotype

Restriction enzyme Nco I was used to digest the PCR products of *ADAMTS1* gene amplified by primer P3, and three genotypes (AA, AG and GG) were identified (Figure 3a). The PCR products of *ADAMTS1* gene amplified by primer P7 were digested by restriction enzyme Hha I, and three genotypes (CC, CT and TT) were identified (Figure 3b).

### 3.3 SNPs identified by sequencing

**FIGURE 4:**
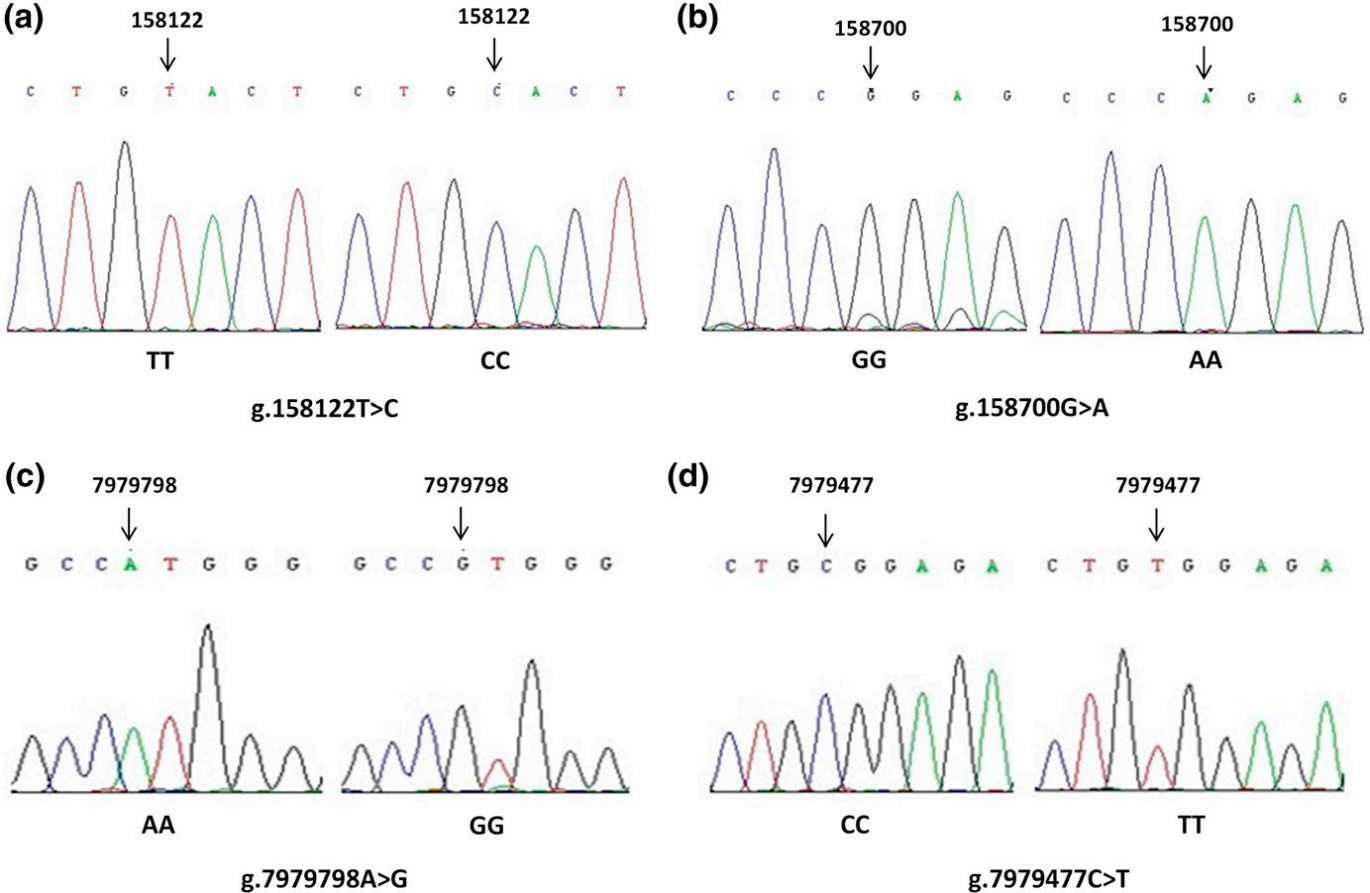
Nucleotide mutations in caprine *ASMT* and *ADAMTS1* genes. (a) Single nucleotide polymorphism (SNP) g.158122T>C in caprine *ASMT* gene. (b) SNP g.158700G>A in caprine *ASMT* gene. (c) SNP g.7979798A>G in caprine *ADAMTS1* gene. (d) SNP g.7979477C>T in caprine *ADAMTS1* gene

The PCR products with different genotypes were sequenced to confirm the mutations. The sequences of different genotypes are shown in Figure 4. One SNP g.158122T>C was found in the sequences amplified with primer P2 of the *ASMT* gene, which was located in exon 8 (Figure 4a). One SNP g.158700G>A was found in the sequences amplified with primer P3 of the *ASMT* gene, which was located in 3′‐regulatory region (Figure 4b). Meanwhile, SNP g.158122T>C caused an amino acid change at residue 298 (Tyr to His, Y to H).

For primer P3 of *ADAMTS1* gene, the sequencing results of three genotypes (AA, AG and GG) revealed one mutation (g.7979798A>G; Figure 4c). For primer P7 of *ADAMTS1* gene, three genotypes (CC, CT and TT) were sequenced, and one mutation (g.7979477C>T) was revealed (Figure 4d).

### 3.4 Allele and genotype frequencies of *ASMT* and *ADAMTS1* genes in five goat breeds

Allele and genotype frequencies of *the ASMT* gene in five goat breeds are presented in Table 3. The results indicated that at g.158122T>C, C allele is dominant allele in Jining Grey goat, but not in Boer goat, Angora goat, and Liaoning Cashmere goat. The homozygotes are not present in Inner Mongolia Cashmere goat, only the heterozygotes exist. g.158122T>C locus was moderately polymorphic (0.25 < PIC < 0.5) in Jining Grey goat, Liaoning Cashmere goat, and Inner Mongolia Cashmere goat, and at a low rate of polymorphism (PIC < 0.25) in Boer goat and Angora goat. Chi‐square test showed that the population of Jining Grey goat and Liaoning Cashmere goat were in a state of Hardy–Weinberg equilibrium (*p* > 0.05). And at g.158700G>A, G allele is dominant allele in all five goat breeds, and chi‐square test showed that the population of five goat breeds were all in Hardy–Weinberg equilibrium (*p* > 0.05). g.158700G>A locus was moderately polymorphic (0.25 < PIC < 0.5) only in Boer goat.

**TABLE 3:** Allele and genotype frequencies of the *ASMT* gene in five goat breeds

Locus	Breed	Number	Genotype frequency			Allele frequency		PIC	HE	NE	*χ* ^2^ test (*p*‐value)
			TT	TC	CC	T	C				
g.158122T>C	Jining Grey goat	296	0.10 (30)	0.49 (146)	0.41 (120)	0.35	0.65	0.45	0.35	1.83	0.3264
	Boer goat	60	0.78 (47)	0.15 (9)	0.07 (4)	0.86	0.14	0.24	0.21	1.32	0.0122
	Angora goat	60	0.93 (56)	0.05 (3)	0.02 (1)	0.96	0.04	0.08	0.08	1.09	0.0151
	Liaoning Cashmere goat	60	0.54 (32)	0.33 (20)	0.13 (8)	0.70	0.30	0.42	0.33	1.72	0.2788
	Inner Mongolia Cashmere goat	44	0.00 (0)	1.00 (44)	0.00 (0)	0.50	0.50	0.50	0.38	2.00	0.0000

			GG	GA	AA	G	A				
g.158700G>A	Jining Grey goat	243	0.79 (193)	0.19 (45)	0.02 (5)	0.89	0.11	0.20	0.18	1.25	0.4828
	Boer goat	52	0.67 (35)	0.33 (17)	0.00 (0)	0.84	0.16	0.27	0.24	1.38	0.3706
	Angora goat	32	0.91 (29)	0.09 (3)	0.00 (0)	0.95	0.05	0.09	0.09	1.10	0.9620
	Liaoning Cashmere goat	81	0.77 (62)	0.23 (19)	0.00 (0)	0.88	0.12	0.21	0.19	1.26	0.4892
	Inner Mongolia Cashmere goat	60	0.98 (59)	0.02 (1)	0.00 (0)	0.99	0.01	0.02	0.02	1.02	0.9979

Note

Number in parentheses represent sample size.

Abbreviations: HE, heterozygosity; NE, effective number of alleles; PIC, polymorphism information content.

Allele and genotype frequencies of *ADAMTS1* gene in five goat breeds are shown in Table 4. The results indicated that g. 7979798A>G locus was moderately polymorphic (0.25 < PIC < 0.5) in all five goat breeds. And chi‐square test showed that the populations of five goat breeds were all in Hardy–Weinberg equilibrium (*p* > 0.05). At g.7979477C>T, C allele is dominant allele in five goat breeds. Homozygotes TT genotype was only present in Jining Grey goat. Chi‐square test showed that the populations of five goat breeds were all in Hardy–Weinberg equilibrium (*p* > 0.05). g.7979477C>T locus was moderately polymorphic (0.25 < PIC < 0.5) only in Guizhou White goat.

**TABLE 4:** Allele and genotype frequencies of the *ADAMTS1* gene in five goat breeds

Locus	Breed	Number	Genotype frequency			Allele frequency		PIC	HE	NE	*χ* ^2^ test (*p*‐value)
			AA	AG	GG	A	G				
g.7979798A>G	Jining Grey goat	243	0.18 (45)	0.78 (189)	0.04 (9)	0.57	0.43	0.37	0.49	1.96	0.0000
	Guizhou White goat	55	0.18 (10)	0.66 (36)	0.16 (9)	0.51	0.49	0.50	0.37	2.00	0.0717
	Boer goat	32	0.22 (7)	0.78 (25)	0.00 (0)	0.61	0.39	0.48	0.36	1.91	0.0014
	Liaoning Cashmere goat	82	0.23 (19)	0.64 (52)	0.13 (11)	0.55	0.45	0.50	0.37	1.98	0.0397
	Inner Mongolia Cashmere goat	60	0.08 (5)	0.70 (42)	0.22 (13)	0.43	0.57	0.49	0.37	1.97	0.0044

			CC	CT	TT	C	T				
g.7979477C>T	Jining Grey goat	243	0.79 (193)	0.19 (45)	0.02 (5)	0.89	0.11	0.20	0.18	1.25	0.4828
	Guizhou White goat	52	0.67 (35)	0.33 (17)	0.00 (0)	0.84	0.16	0.27	0.24	1.38	0.3706
	Boer goat	32	0.91 (29)	0.09 (3)	0.00 (0)	0.95	0.05	0.09	0.09	1.10	0.9620
	Liaoning Cashmere goat	81	0.77 (62)	0.23 (19)	0.00 (0)	0.88	0.12	0.21	0.19	1.26	0.4892
	Inner Mongolia Cashmere goat	60	0.98 (59)	0.02 (1)	0.00 (0)	0.99	0.01	0.02	0.02	1.02	0.9979

Note

Number in parentheses represent sample size.

Abbreviations: HE, heterozygosity; NE, effective number of alleles; PIC, polymorphism information content.

### 3.5 Influence of different genotypes on litter size in Jining Grey goats

The least squares means and standard error for litter size of different *ASMT* genotypes in Jining Grey goats are presented in Table 5. For g.158122T>C of *ASMT* gene, the Jining Grey goat does with genotype TC and CC had 0.66 (*p* < 0.05) and 0.75 (*p* < 0.05) kids more than those with genotype TT respectively. No significant difference (*p* > 0.05) was found in litter size between TC and CC genotypes in Jining Grey goats. For g.158700G>A, no significant difference (*p* > 0.05) was found in litter size between GG, GA and AA genotypes in Jining Grey goats.

**TABLE 5:** Least squares mean and standard error for litter size of different genotypes of the *ASMT* gene in Jining Grey goats

Locus	Genotype	Number of does	Litter size
g.158122T>C	TT	30	1.76^b^ ± 0.19
	TC	146	2.42^a^ ± 0.14
	CC	120	2.51^a^ ± 0.15
g.158700G>A	GG	110	2.45^a^ ± 0.17
	GA	170	2.36^a^ ± 0.14
	AA	16	2.29^a^ ± 0.20

Note

Means within the same group with different superscripts are significantly different (*p* < 0.05).

Table 6 shows the least squares means and standard error for litter size of different *ADAMTS1* genotypes in Jining Grey goats. For SNP g.7979798A>G, there was no significant difference (*p* > 0.05) in litter size of different genotypes in Jining Grey goats. Regarding SNP g.7979477C>T, the Jining Grey goats with genotype CT or TT had 0.82 (*p* < 0.05) or 0.86 (*p* < 0.05) more kids than those with genotype CC. No significant difference (*p* > 0.05) was found in litter size between TT and CT genotypes.

**TABLE 6:** Least squares mean and standard error for litter size of different genotypes of the *ADAMTS1* gene in Jining Grey goats

Locus	Genotype	Number of does	Litter size
52.**8**g.7979798A>G	AA	45	2.40^a^ ± 0.15
	AG	189	2.29^a^ ± 0.12
	GG	9	2.01^a^ ± 0.19
g.7979477C>T	CC	193	2.13^b^ ± 0.07
	CT	45	2.95^a^ ± 0.11
	TT	5	2.99^a^ ± 0.14

Note

Means within the same group with different superscripts are significantly different (*p* < 0.05).

### 3.6 Bioinformatics analysis of *ASMT* gene with SNP g.158122T>C

**FIGURE 5:**
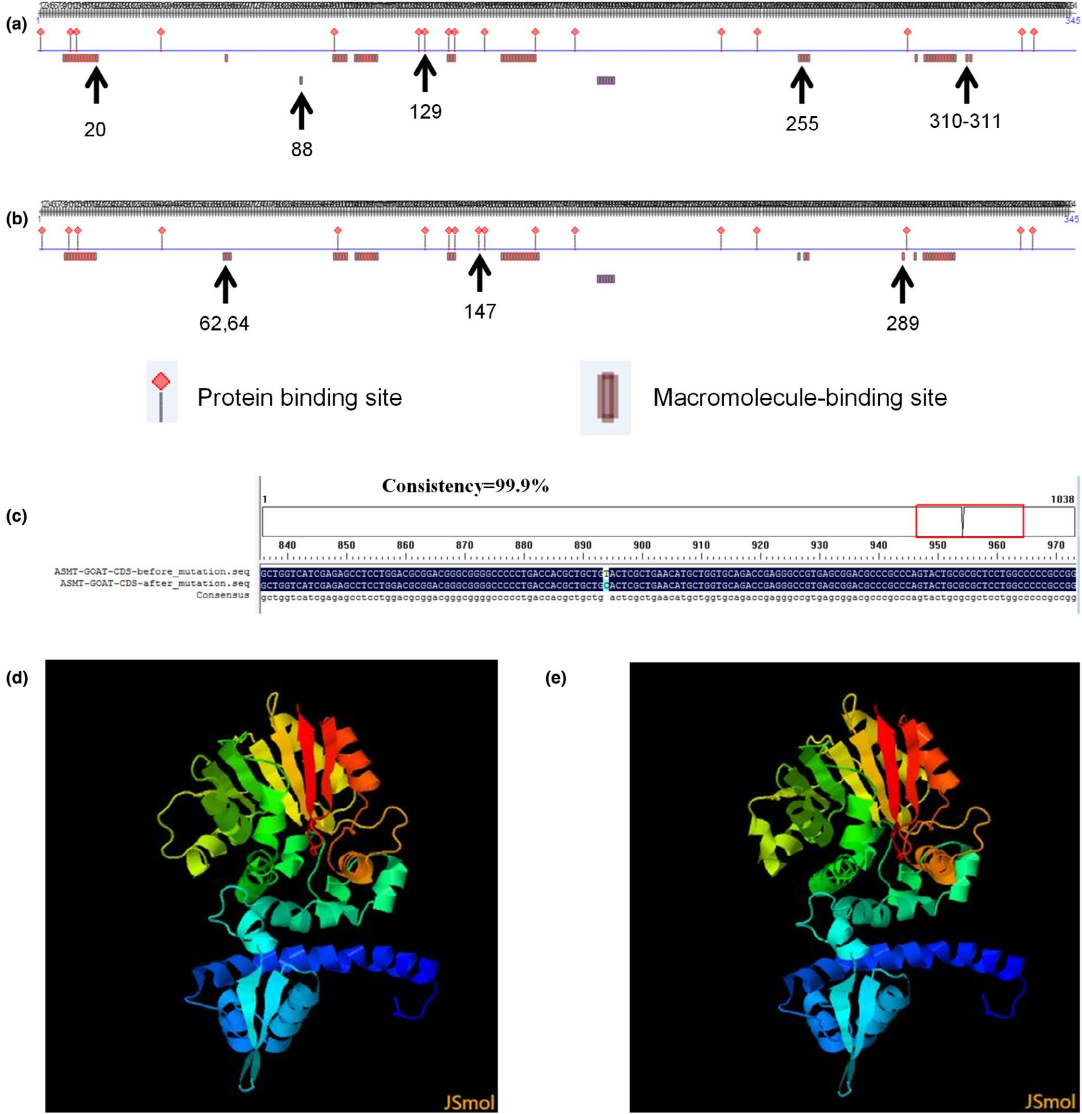
Secondary structure and tertiary structure of ASMT protein before and after the mutation at g.158122T>C based on its predicted amino acid sequence. (a) Protein secondary structure before the mutation (T Allele). (b) Secondary protein structure after the mutation (C Allele). (c) CDS sequence alignment before and after mutation. (d) The 3‐dimension structure prediction before mutation (T Allele). (e) The 3‐dimension structure prediction after mutation (C Allele)

SNP g.158122T>C (Figure 5c) of *ASMT* caused an amino acid (AA) change at residue 298 (Tyr to His, Y to H). The protein secondary structure before and after mutation at g.158122T>C was also predicted by PreditProtein. Compared with the wild‐type allele (T) and the mutant allele (C) caused one protein binding site (AA 129) and five macromolecule‐binding sites (AA 20, 88, 255, 310, 311) to be lost, and alsoobtain a new protein binding site (AA 147) and three new macromolecule‐binding sites (AA 62, 64, 289; Figure 5a,b). The 3‐dimension structure before and after mutation in ASMT were predicted via Phyre2, it can be observed that the tertiary structure of the protein changed significantly before and after mutation (Figure 5d,e).

## 4 DISCUSSION

### 4.1 *ASMT* gene

#### 4.1.1 Association between *ASMT* and reproductive performance

ASMT is the last step key enzyme for catalysis of 5‐serotonin to MLT and MLT plays a crucial role in the regulation of animal reproductive processes directly (El‐Raey et al., 2011; Iwasaki et al., 2005; Lanoix, Beghdadi, Lafond, & Vaillancourt, 2008) and indirectly (GnRH production in the hypothalamus). *ASMT* gene may be involved in multiple functions including reproductive regulation.

In early research, *ASMT* mRNA was detected in the pineal gland, retina and ovary in rat (Gauer & Craft, 1996; Itoh et al., 1997), as well as oocyte, blastoderm and ovarian follicles in Japanese quail (Olszanska, Majewski, Lewczuk, & Stepinska, 2007). *ASMT* gene in the testes of rat expressed with 24‐hr rhythms and reached the maximal values during the dark phase (Coelho et al., 2019). But the mRNA levels and enzyme activities of the ASMT and endogenous MLT displayed no significant variation throughout the oestrous cycle of rat (Skorupa et al., 2003) From sequencing data, it showed *ASMT* mRNA has high expression level in the ovary, testis, adrenal and mammary gland in mouse (Yue et al., 2014). In human, *ASMT* mRNA has the highest expression level in the ovary (Fagerberg et al., 2014). The enzyme activity of ASMT and MLT were higher in gestation‐matched normotensive controls than the preeclamptic pregnancy placentas, despite insignificant expression difference for both transcript and protein of ASMT in placentas between the affected and controls (Lanoix, Guerin, & Vaillancourt, 2012). In sheep, it has a high expression level in skin, kidney, testes, and hypothalamus (Jiang et al., 2014). A higher concentration of MLT was found in ram seminal plasma than that in blood, gene expression of *ASMT* was high in the testis, and ASMT protein in the testis was found in the Leydig cells, spermatocytes, and spermatids (Gonzalez‐Arto et al., 2016).. Furthermore, our previous study showed that *ASMT* mRNA expressed dramatically distinctive between Jining Grey goat and non‐prolific Liaoning Cashmere goat (Huang., 2012).

#### 4.1.2 Polymorphisms of *ASMT* gene

Polymorphisms of the *ASMT* gene can be one important cause for the significant change of enzyme activity in humans (Chaste et al., 2011; Etain et al., 2012; Pagan et al., 2011). A large number of mutations of human *ASMT* gene have been screened to determine candidate alleles with some mental diseases, such as intellectual disability (Pagan et al., 2011), attention‐deficit/hyperactivity disorder (Chaste et al., 2011), bipolar disorder (Etain et al., 2012), autism (Jonsson et al., 2010; Wang et al., 2013). A polymorphism (rs4446909) of the promoter of the *ASMT* gene associated with bipolar disorder influences sleep and circadian rhythms, and it associated with lower *ASMT* transcription level and weaker activity in lymphoblastoid cell lines (Geoffroy et al., 2014). More than ten nonsynonymous variants of *ASMT* identified through the 1,000 genomes project, stopped or reduced ASMT activity in patients with neuropsychiatric disorders, and one of these variants (N17K, rs17149149) is a relatively frequent polymorphism in the Han Chinese population (Botros et al., 2013).

A restriction enzyme BglⅡ site in intron 8 of the chicken *ASMT* gene was found (Grechez‐Cassiau, Bernard, Ladjali, Rodriguez, & Voisin, 1998). Two mutations in exon 5 (G606A) and exon 6 (A737C) of sheep *ASMT* gene were identified, which was unassociated with seasonal reproduction and litter size (Sun., 2013). However, polymorphism of the goat *ASMT* gene was rare reported, the association of polymorphism with reproduction in goats was unclear.

The present study found two SNPs and one (g.158122T>C) was located in the coding region and the other (g.158700G>A) was in 3′‐regulatory region of the goat *ASMT* gene. For g.158122T>C, the genotype distribution was different between prolific Jining Grey goat and the other four non‐prolific goat breeds, and C allele frequency in Jining Grey goat was higher than other breeds with less litter size. g.158122T>C locus was moderately polymorphic (0.25 < PIC < 0.5) in Jining Grey goat, it indicated that the locus had strong selection potential in these sheep populations. And the Jining Grey goats with genotype CC and TC had more litter size than those with TT for g.158122T>C. And SNP g.158122T>C caused an amino acid change from weakly polar aromatic Tyr to polar basic His, in which amino acid residue His may participate in the enzyme activity centre. And this mutation would cause a protein binding site and five macromolecule‐binding sites lost, and also obtain a new protein binding site and three new macromolecule‐binding sites. The improvement in reproductive performance may be due to changes in the structure of the ASMT protein.

So we speculated that the C allele of g.158122T>C locus may be one of the causal candidates for prolificacy in Jining Grey goat. The selection of individuals with CC would be more profitable. Further study should validate the association study, given that TT individuals were fewer than TC or CC.

### 4.2 *ADAMTS1* gene

#### 4.2.1 Association between *ADAMTS1* and reproductive performance

As a multifunctional protease, ADAMTS1 is capable of cleaving matrix proteoglycans such as aggrecan, versican and brevican. During Drosophila oogenesis, ADAMTS1 motif A (AdamTS‐A) was identified as a novel target of Janus kinase/signal transducer and activator of transcription (JAK/STAT) in epithelial follicle cells which regulates egg chamber shape by remodelling the basement membrane(Wittes & Schupbach, 2019). To the female mice which lack *ADAMTS1*, mature oocytes trapped in the follicles, resulted in impaired ovulation and subfertility (Mittaz et al., 2004). *ADAMTS1* null ovaries had some unusual atretic follicles (Shozu et al., 2005). The morphological assessment revealed peri‐ovulatory ovaries had abnormal morphogenesis (Brown et al., 2010). In ovulation, *ADAMTS1* could cleave versican in the mature COC matrix. However, the *ADAMTS1* null mice lost this function partially (Russell, Doyle, Ochsner, Sandy, & Richards, 2003). Ovulation rate reduced 77%, and the fertilization rate of oocytes reduced about 63% in *ADAMTS1* null mice, it caused reduced litter size and littered number. Shindo et al. (2000) also found *ADAMTS1* null mice had a significantly low number of pups and delivery rate, it suggested *ADAMTS1* null female mice were infertile. It also reported that the number of implantation sites was decreased in *ADAMTS1* null mice (Mittaz et al., 2004; Shindo et al., 2000), and loss of the mature form of ADAMTS1 caused the developmental arrest of early secondary follicles (Meng et al., 2017). The newest research also indicated ADAMTS family members play important roles in follicle rupture in cattle (Willis et al., 2017).

#### 4.2.2 Polymorphisms of *ADAMTS1*gene

Current studies have found that the polymorphism of the *ADAMTS1* gene is associated with many diseases. Polymorphisms of the *ADAMTS1* gene (rs416905 and rs402007) may be associated with ischemic stroke caused by arge artery atherosclerosis (Lyu et al., 2015). Two SNPs (rs2738, rs229038) of ADAMTS1 were significantly associated with mandibular prognathism (Guan et al., 2015; Liu et al., 2017). One SNP of ADAMTS1 (rs12140) decreased the risk of dying from colorectal cancer (Mullany, Herrick, Wolff, & Slattery, 2017). Polymorphisms within the *ADAMTS1* gene influenced the effectiveness of a drug called statins in reducing the risk of myocardial infarction, homozygous of ADAMTS1 rs402007 had the most benefit from statins (Peters et al., 2010).

The polymorphism of the *ADAMTS1* gene is abundant. An SNP of the *ADAMTS1* gene *was* found in Landrace pig by RFLP and correlation analysis indicated that this SNP was significantly correlated with litter size and number born alive (Shan‐Shan, 2008). Two mutations in pig *ADAMTS1* gene consisting of one C72G mutation in exon 7 and one G512A mutation in intron 7 were detected, of which the former one caused an arginine to proline change at position 622 (Arg622Pro). New Qingping sows with heterozygote genotype GC (for SNP C72G) or GA (for SNP G512A) had more litter size and live litter size than other genotypes (Le., 2008). There are 513 SNP loci of goat *ADAMTS1* gene published in Ensembl database. However, studies on the relationship between *ADAMTS1* gene polymorphism and reproduction are rare.

In our study, for g.7979477C>T of *ADAMTS1* gene, it was moderately polymorphic (0.25 < PIC < 0.5) only in Guizhou White goat, it indicated that the locus had strong selection potential in these goat populations. Nevertheless, Jining Grey goat, Boer goat, Liaoning Cashmere goat, and Inner Mongolia Cashmere goat had low polymorphism (PIC < 0.25), it indicated that the genetic diversity of this locus was relatively poor in these four goat breeds. But the populations of five goat breeds were all in Hardy‐Weinberg equilibrium (*p* > 0.05). C allele is a dominant allele in all five goat breeds. Homozygotes TT genotype was only present in Jining Grey goat. It may be related to the small sample size of goats selected in this study. Jining Grey goats with genotype CT or TT had 0.82 (*p* < 0.05) or 0.86 (*p* < 0.05) more kids than those with genotype CC respectively. The Jining Grey goats with genotype TT had 0.04 (*p* > 0.05) more kids than those with genotype CT. So we speculated that T allele of g.7979477C>T locus may be one of the causal candidates for prolificacy in Jining Grey goat. The selection of individuals with TT would be more profitable. In goats, the T allele at the g.7979477C>T locus of *ADAMTS1* gene could be a potential marker for improving litter size of goat.

## 5 CONCLUSION

In this study, two SNPs in goat *ASMT* gene and two SNPs in goat *ADAMTS1* gene were identified. The results indicated that C allele of the g.158122T>C locus of *ASMT* gene and the T allele at the g.7979477C>T locus of *ADAMTS1* gene were potential molecular markers which could improve litter size of Jining Grey goats and be used in goat breeding. This novel mutation provides further evidence that *ASMT* and *ADAMTS1* gene may play key roles in reproductive function.

## 6 ETHICS APPROVAL AND CONSENT TO PARTICIPATE

All procedures involving animals were authorized and approved by the Animal Ethics Committee of the Institute of Animal Science, Chinese Academy of Agricultural Sciences with the following number: IASCAAS‐AE‐03.

## CONFLICT OF INTEREST

The authors declare no conflict of interest. The founding sponsors had no role in the design of the study; in the collection, analyses, or interpretation of data; in the writing of the manuscript, and in the decision to publish the results.

## AUTHOR CONTRIBUTION

**Wenping Hu:** Conceptualization; Data curation; Formal analysis; Funding acquisition; Project administration; Supervision; Validation; Visualization; Writing‐original draft; Writing‐review & editing. **Jishun Tang:** Data curation; Software; Validation; Visualization; Writing‐review & editing. **Zhuangbiao Zhang:** Methodology; Software; Visualization. **Qianqian Tang:** Investigation. **Yan Yan:** Investigation. **PingQing Wang:** Methodology. **Xiangyu Wang:** Methodology; Writing‐review & editing. **Qiuyue Liu:** Writing‐review & editing. **Xiaofei Guo:** Methodology. **Mei Jin:** Methodology. **Yingjie Zhang:** Methodology. **Ran Di:** Conceptualization; Supervision. **Mingxing Chu:** Conceptualization; Funding acquisition; Project administration; Supervision.

## Data Availability

All public data generated or analyzed during this study are included in this article. Data sharing is not applicable to this article as no new data were created or analyzed in this study.

## References

[vms3301-bib-0001] Aydos, O. S. , Yukselten, Y. , Ozkavukcu, S. , Sunguroglu, A. , & Aydos, K. (2019). ADAMTS1 and ADAMTS5 metalloproteases produced by Sertoli cells: A potential diagnostic marker in azoospermia. Systems Biology in Reproductive Medicine, 65, 29–38. 10.1080/19396368.2018.1467512 29737873

[vms3301-bib-0002] Boerboom, D. , Russell, D. L. , Richards, J. S. , & Sirois, J. (2003). Regulation of transcripts encoding ADAMTS‐1 (a disintegrin and metalloproteinase with thrombospondin‐like motifs‐1) and progesterone receptor by human chorionic gonadotropin in equine preovulatory follicles. Journal of Molecular Endocrinology, 31, 473–485. 10.1677/jme.0.0310473 14664708

[vms3301-bib-0003] Botros, H. G. , Legrand, P. , Pagan, C. , Bondet, V. , Weber, P. , Ben‐Abdallah, M. , … Bourgeron, T. (2013). Crystal structure and functional mapping of human ASMT, the last enzyme of the melatonin synthesis pathway. Journal of Pineal Research, 54, 46–57. 10.1111/j.1600-079X.2012.01020.x 22775292

[vms3301-bib-0004] Brown, H. M. , Dunning, K. R. , Robker, R. L. , Boerboom, D. , Pritchard, M. , Lane, M. , & Russell, D. L. (2010). ADAMTS1 cleavage of versican mediates essential structural remodeling of the ovarian follicle and cumulus‐oocyte matrix during ovulation in mice. Biology of Reproduction, 83, 549–557. 20592310 10.1095/biolreprod.110.084434

[vms3301-bib-0005] Brown, H. M. , & Russell, D. L. (2014). Blood and lymphatic vasculature in the ovary: Development, function and disease. Human Reproduction Update, 20, 29–39. 10.1093/humupd/dmt049 24097804

[vms3301-bib-0006] Byeon, Y. , & Back, K. (2016). Melatonin production in Escherichia coli by dual expression of serotonin N‐acetyltransferase and caffeic acid O‐methyltransferase. Applied Microbiology and Biotechnology, 100, 6683–6691. 10.1007/s00253-016-7458-z 27005412

[vms3301-bib-0007] Chaste, P. , Clement, N. , Botros, H. G. , Guillaume, J. L. , Konyukh, M. , Pagan, C. , … Bourgeron, T. (2011). Genetic variations of the melatonin pathway in patients with attention‐deficit and hyperactivity disorders. Journal of Pineal Research, 51, 394–399. 10.1111/j.1600-079X.2011.00902.x 21615493

[vms3301-bib-0008] Coelho, L. A. , Andrade‐Silva, J. , Motta‐Teixeira, L. C. , Amaral, F. G. , Reiter, R. J. , & Cipolla‐Neto, J. (2019). The absence of pineal melatonin abolishes the daily rhythm of Tph1 (tryptophan hydroxylase 1), Asmt (acetylserotonin o‐methyltransferase), and Aanat (aralkylamine N‐acetyltransferase) mRNA expressions in rat testes. Molecular Neurobiology, 56, 7800–7809. 10.1007/s12035-019-1626-y 31124080

[vms3301-bib-0009] Dollins, A. B. , Zhdanova, I. V. , Wurtman, R. J. , Lynch, H. J. , & Deng, M. H. (1994). Effect of inducing nocturnal serum melatonin concentrations in daytime on sleep, mood, body temperature, and performance. Proceedings of the National Academy of Sciences of the United States of America, 91(5), 1824–1828. 10.1073/pnas.91.5.1824 8127888 PMC43256

[vms3301-bib-0010] Dunlap, K. A. , Kwak, H. I. , Burghardt, R. C. , Bazer, F. W. , Magness, R. R. , Johnson, G. A. , & Bayless, K. J. (2010). The sphingosine 1‐phosphate (S1P) signaling pathway is regulated during pregnancy in sheep. Biology of Reproduction, 82, 876–887. 20107206 10.1095/biolreprod.109.081604 PMC2857631

[vms3301-bib-0011] El‐Raey, M. , Geshi, M. , Somfai, T. , Kaneda, M. , Hirako, M. , Abdel‐Ghaffar, A. E. , … Nagai, T. (2011). Evidence of melatonin synthesis in the cumulus oocyte complexes and its role in enhancing oocyte maturation in vitro in cattle. Molecular Reproduction and Development, 78, 250–262. 10.1002/mrd.21295 21381146

[vms3301-bib-0012] Espey, L. L. , Yoshioka, S. , Russell, D. L. , Robker, R. L. , Fujii, S. , & Richards, J. S. (2000). Ovarian expression of a disintegrin and metalloproteinase with thrombospondin motifs during ovulation in the gonadotropin‐primed immature rat. Biology of Reproduction, 62, 1090–1095. 10727282 10.1095/biolreprod62.4.1090

[vms3301-bib-0013] Etain, B. , Dumaine, A. , Bellivier, F. , Pagan, C. , Francelle, L. , Goubran‐Botros, H. , … Jamain, S. (2012). Genetic and functional abnormalities of the melatonin biosynthesis pathway in patients with bipolar disorder. Human Molecular Genetics, 21, 4030–4037. 10.1093/hmg/dds227 22694957

[vms3301-bib-0014] Fagerberg, L. , Hallstrom, B. M. , Oksvold, P. , Kampf, C. , Djureinovic, D. , Odeberg, J. , … Uhlen, M. (2014). Analysis of the human tissue‐specific expression by genome‐wide integration of transcriptomics and antibody‐based proteomics. Molecular & Cellular Proteomics, 13, 397–406. 24309898 10.1074/mcp.M113.035600 PMC3916642

[vms3301-bib-0015] Gauer, F. , & Craft, C. M. (1996). Circadian regulation of hydroxyindole‐O‐methyltransferase mRNA levels in rat pineal and retina. Brain Research, 737, 99–109. 10.1016/0006-8993(96)00632-4 8930356

[vms3301-bib-0016] Geoffroy, P. A. , Boudebesse, C. , Henrion, A. , Jamain, S. , Henry, C. , Leboyer, M. , … Etain, B. (2014). An ASMT variant associated with bipolar disorder influences sleep and circadian rhythms: A pilot study. Genes, Brain, and Behavior, 13, 299–304. 10.1111/gbb.12103 24308489

[vms3301-bib-0017] Gonzalez‐Arto, M. , Hamilton, T. R. , Gallego, M. , Gaspar‐Torrubia, E. , Aguilar, D. , Serrano‐Blesa, E. , … Casao, A. (2016). Evidence of melatonin synthesis in the ram reproductive tract. Andrology, 4, 163–171. 10.1111/andr.12117 26742835

[vms3301-bib-0018] Grechez‐Cassiau, A. , Bernard, M. , Ladjali, K. , Rodriguez, I. R. , & Voisin, P. (1998). Structural analysis of the chicken hydroxyindole‐O‐methyltransferase gene. European Journal of Biochemistry, 258, 44–52. 10.1046/j.1432-1327.1998.2580044.x 9851690

[vms3301-bib-0019] Guan, X. , Song, Y. , Ott, J. , Zhang, Y. , Li, C. , Xin, T. , … Zhou, Y. (2015). The *ADAMTS1* gene is associated with familial mandibular prognathism. Journal of Dental Research, 94, 1196–1201. 26124221 10.1177/0022034515589957

[vms3301-bib-0020] He, X. , Li, B. , Wang, F. , Tian, C. , Rong, W. , & Liu, Y. (2012). Identification of differentially expressed genes in Mongolian sheep ovaries by suppression subtractive hybridization. Animal Reproduction Science, 133, 86–92. 22727452 10.1016/j.anireprosci.2012.06.005

[vms3301-bib-0021] Hirohata, S. , Inagaki, J. , & Ohtsuki, T. (2017). Diverse functions of a disintegrin and metalloproteinase with thrombospondin motif‐1. Yakugaku Zasshi: Journal of the Pharmaceutical Society of Japan, 137, 811–814. 28674292 10.1248/yakushi.16-00236-4

[vms3301-bib-0022] Huang, D. W. (2012). Study on cloning, polymorphisms and expression of genes related with seasonal reproduction pathways in goats. Doctoral degree, Chinese Academy of Agricultural Sciences, China.

[vms3301-bib-0023] Itoh, M. T. , Ishizuka, B. , Kudo, Y. , Fusama, S. , Amemiya, A. , & Sumi, Y. (1997). Detection of melatonin and serotonin N‐acetyltransferase and hydroxyindole‐O‐methyltransferase activities in rat ovary. Molecular and Cellular Endocrinology, 136, 7–13. 9510062 10.1016/s0303-7207(97)00206-2

[vms3301-bib-0024] Iwasaki, S. , Nakazawa, K. , Sakai, J. , Kometani, K. , Iwashita, M. , Yoshimura, Y. , & Maruyama, T. (2005). Melatonin as a local regulator of human placental function. Journal of Pineal Research, 39, 261–265. 16150106 10.1111/j.1600-079X.2005.00244.x

[vms3301-bib-0025] Jiang, Y. , Xie, M. , Chen, W. , Talbot, R. , Maddox, J. F. , Faraut, T. , … Dalrymple, B. P. (2014). The sheep genome illuminates biology of the rumen and lipid metabolism. Science, 344, 1168–1173. 24904168 10.1126/science.1252806 PMC4157056

[vms3301-bib-0026] Jonsson, L. , Ljunggren, E. , Bremer, A. , Pedersen, C. , Landen, M. , Thuresson, K. , … Melke, J. (2010). Mutation screening of melatonin‐related genes in patients with autism spectrum disorders. BMC Medical Genomics, 3, 10. 20377855 10.1186/1755-8794-3-10 PMC3020629

[vms3301-bib-0027] Karakose, M. , Demircan, K. , Tutal, E. , Demirci, T. , Arslan, M. S. , Sahin, M. , … Delibasi, T. (2016). Clinical significance of ADAMTS1, ADAMTS5, ADAMTS9 aggrecanases and IL‐17A, IL‐23, IL‐33 cytokines in polycystic ovary syndrome. Journal of Endocrinological Investigation, 39, 1269–1275. 27146815 10.1007/s40618-016-0472-2

[vms3301-bib-0028] Kunkle, B. W. , Grenier‐Boley, B. , Sims, R. , Bis, J. C. , Damotte, V. , Naj, A. C. , … Pericak‐Vance, M. A. (2019). Genetic meta‐analysis of diagnosed Alzheimer's disease identifies new risk loci and implicates Abeta, tau, immunity and lipid processing. Nature Genetics, 51, 414–430. 30820047 10.1038/s41588-019-0358-2 PMC6463297

[vms3301-bib-0029] Kuno, K. , Okada, Y. , Kawashima, H. , Nakamura, H. , Miyasaka, M. , Ohno, H. , & Matsushima, K. (2000). ADAMTS‐1 cleaves a cartilage proteoglycan, aggrecan. FEBS Letters, 478, 241–245. 10930576 10.1016/s0014-5793(00)01854-8

[vms3301-bib-0030] Lanoix, D. , Beghdadi, H. , Lafond, J. , & Vaillancourt, C. (2008). Human placental trophoblasts synthesize melatonin and express its receptors. Journal of Pineal Research, 45, 50–60. 18312298 10.1111/j.1600-079X.2008.00555.x

[vms3301-bib-0031] Lanoix, D. , Guerin, P. , & Vaillancourt, C. (2012). Placental melatonin production and melatonin receptor expression are altered in preeclampsia: New insights into the role of this hormone in pregnancy. Journal of Pineal Research, 53, 417–425. 10.1111/j.1600-079X.2012.01012.x 22686298

[vms3301-bib-0032] Le, K. (2008). Cloning, radiation hybrid mapping, promoter regulation and genetics effect analysis of procine *ADAMTS1* gene. Doctoral degree, Huazhong Agricultural University, China.

[vms3301-bib-0033] Liu, H. , Wu, C. , Lin, J. , Shao, J. , Chen, Q. , & Luo, E. (2017). Genetic etiology in nonsyndromic mandibular prognathism. The Journal of Craniofacial Surgery, 28, 161–169. 10.1097/SCS.0000000000003287 27941554

[vms3301-bib-0034] Lussier, J. G. , Diouf, M. N. , Levesque, V. , Sirois, J. , & Ndiaye, K. (2017). Gene expression profiling of upregulated mRNAs in granulosa cells of bovine ovulatory follicles following stimulation with hCG. Reproductive Biology and Endocrinology, 15, 88. 29100496 10.1186/s12958-017-0306-x PMC5670713

[vms3301-bib-0035] Lyu, C. , Chen, Y. , Zhu, M. , Jin, X. , Liu, P. , Zheng, Z. , … Wang, W. (2015). Association of ADAMTS‐1 gene polymorphisms with ischemic stroke caused by large artery atherosclerosis. Chinese Journal of Medical Genetics, 32, 844–848. 26663063 10.3760/cma.j.issn.1003-9406.2015.06.021

[vms3301-bib-0036] Malan, S. W. (2000). The improved Boer goat. Small Ruminant Research, 36, 165–170. 10760452 10.1016/s0921-4488(99)00160-1

[vms3301-bib-0037] Meng, T. G. , Hu, M. W. , Ma, X. S. , Huang, L. , Liang, Q. X. , Yuan, Y. , … Sun, Q. Y. (2017). Oocyte‐specific deletion of furin leads to female infertility by causing early secondary follicle arrest in mice. Cell Death & Disease, 8, e2846 10.1038/cddis.2017.231 28569793 PMC5520891

[vms3301-bib-0038] Mishra, B. , Koshi, K. , Kizaki, K. , Ushizawa, K. , Takahashi, T. , Hosoe, M. , … Hashizume, K. (2013). Expression of ADAMTS1 mRNA in bovine endometrium and placenta during gestation. Domestic Animal Endocrinology, 45, 43–48. 10.1016/j.domaniend.2013.04.002 23751571

[vms3301-bib-0039] Mittaz, L. , Russell, D. L. , Wilson, T. , Brasted, M. , Tkalcevic, J. , Salamonsen, L. A. , … Pritchard, M. A. (2004). Adamts‐1 is essential for the development and function of the urogenital system. Biology of Reproduction, 70, 1096–1105. 14668204 10.1095/biolreprod.103.023911

[vms3301-bib-0040] Mullany, L. E. , Herrick, J. S. , Wolff, R. K. , & Slattery, M. L. (2017). Single nucleotide polymorphisms within MicroRNAs, MicroRNA targets, and MicroRNA biogenesis genes and their impact on colorectal cancer survival. Genes, Chromosomes & Cancer, 56, 285–295. 10.1002/gcc.22434 27859935 PMC6007859

[vms3301-bib-0041] Olszanska, B. , Majewski, P. , Lewczuk, B. , & Stepinska, U. (2007). Melatonin and its synthesizing enzymes (arylalkylamine N‐acetyltransferase‐like and hydroxyindole‐O‐methyltransferase) in avian eggs and early embryos. Journal of Pineal Research, 42, 310–318. 17349030 10.1111/j.1600-079X.2007.00421.x

[vms3301-bib-0042] Pagan, C. , Botros, H. G. , Poirier, K. , Dumaine, A. , Jamain, S. , Moreno, S. , … Bourgeron, T. (2011). Mutation screening of ASMT, the last enzyme of the melatonin pathway, in a large sample of patients with intellectual disability. BMC Medical Genetics, 12, 17 10.1186/1471-2350-12-17 21251267 PMC3034665

[vms3301-bib-0043] Peters, B. J. , Rodin, A. S. , Klungel, O. H. , Stricker, B. H. , de Boer, A. , & Maitland‐van der Zee, A. H. (2010). Variants of ADAMTS1 modify the effectiveness of statins in reducing the risk of myocardial infarction. Pharmacogenetics and Genomics, 20, 766–774. 10.1097/FPC.0b013e328340aded 21037509

[vms3301-bib-0044] Recio, J. , Mediavilla, M. D. , Cardinali, D. P. , & Sanchez‐Barcelo, E. J. (1994). Pharmacological profile and diurnal rhythmicity of 2‐[125I]‐iodomelatonin binding sites in murine mammary tissue. Journal of Pineal Research, 16, 10–17. 8158518 10.1111/j.1600-079x.1994.tb00076.x

[vms3301-bib-0045] Roberts, A. J. , & Reeves, J. J. (1988). Kidding rates of angora goats passively immunized against estrogens. Journal of Animal Science, 66, 2443–2447. 10.2527/jas1988.66102443x 3198526

[vms3301-bib-0046] Robker, R. L. , Russell, D. L. , Espey, L. L. , Lydon, J. P. , O'Malley, B. W. , & Richards, J. S. (2000). Progesterone‐regulated genes in the ovulation process: ADAMTS‐1 and cathepsin L proteases. Proceedings of the National Academy of Sciences of the United States of America, 97, 4689–4694. 10.1073/pnas.080073497 10781075 PMC18294

[vms3301-bib-0047] Rodriguez‐Manzaneque, J. C. , Westling, J. , Thai, S. N. , Luque, A. , Knauper, V. , Murphy, G. , … Iruela‐Arispe, M. L. (2002). ADAMTS1 cleaves aggrecan at multiple sites and is differentially inhibited by metalloproteinase inhibitors. Biochemical and Biophysical Research Communications, 293, 501–508. 12054629 10.1016/S0006-291X(02)00254-1

[vms3301-bib-0048] Russell, D. L. , Doyle, K. M. , Ochsner, S. A. , Sandy, J. D. , & Richards, J. S. (2003). Processing and localization of ADAMTS‐1 and proteolytic cleavage of versican during cumulus matrix expansion and ovulation. The Journal of Biological Chemistry, 278, 42330–42339. 10.1074/jbc.M300519200 12907688

[vms3301-bib-0049] Sandy, J. D. , Westling, J. , Kenagy, R. D. , Iruela‐Arispe, M. L. , Verscharen, C. , Rodriguez‐Mazaneque, J. C. , … Clowes, A. W. (2001). Versican V1 proteolysis in human aorta in vivo occurs at the Glu441‐Ala442 bond, a site that is cleaved by recombinant ADAMTS‐1 and ADAMTS‐4. The Journal of Biological Chemistry, 276, 13372–13378. 11278559 10.1074/jbc.M009737200

[vms3301-bib-0050] Sayasith, K. , Lussier, J. , & Sirois, J. (2013). Molecular characterization and transcriptional regulation of a disintegrin and metalloproteinase with thrombospondin motif 1 (ADAMTS1) in bovine preovulatory follicles. Endocrinology, 154, 2857–2869. 10.1210/en.2013-1140 23751874

[vms3301-bib-0051] Schuermann, Y. , Rovani, M. T. , Gasperin, B. , Ferreira, R. , Ferst, J. , Madogwe, E. , … Duggavathi, R. (2018). ERK1/2‐dependent gene expression in the bovine ovulating follicle. Scientific Reports, 8, 16170 10.1038/s41598-018-34015-4 30385793 PMC6212447

[vms3301-bib-0052] Shan‐Shan, X. U. (2008). Genetic effects of ADAMTS‐1 gene on reproductive traits in landrace pig. Journal of Anhui Agricultural Sciences, 36, 10374–10376.

[vms3301-bib-0053] Shindo, T. , Kurihara, H. , Kuno, K. , Yokoyama, H. , Wada, T. , Kurihara, Y. , … Matsushima, K. (2000). ADAMTS‐1: A metalloproteinase‐disintegrin essential for normal growth, fertility, and organ morphology and function. The Journal of Clinical Investigation, 105, 1345–1352. 10.1172/JCI8635 10811842 PMC315464

[vms3301-bib-0054] Shozu, M. , Minami, N. , Yokoyama, H. , Inoue, M. , Kurihara, H. , Matsushima, K. , & Kuno, K. (2005). ADAMTS‐1 is involved in normal follicular development, ovulatory process and organization of the medullary vascular network in the ovary. Journal of Molecular Endocrinology, 35, 343–355. 10.1677/jme.1.01735 16216914

[vms3301-bib-0055] Skorupa, A. L. , Garidou, M. L. , Bothorel, B. , Saboureau, M. , Pevet, P. , Neto, J. C. , & Simonneaux, V. (2003). Pineal melatonin synthesis and release are not altered throughout the estrous cycle in female rats. Journal of Pineal Research, 34, 53–59. 10.1034/j.1600-079X.2003.02952.x 12485372

[vms3301-bib-0056] Soliman, A. , Lacasse, A. A. , Lanoix, D. , Sagrillo‐Fagundes, L. , Boulard, V. , & Vaillancourt, C. (2015). Placental melatonin system is present throughout pregnancy and regulates villous trophoblast differentiation. Journal of Pineal Research, 59, 38–46. 10.1111/jpi.12236 25833399

[vms3301-bib-0057] Sun, X. D. (2013). Studies on the cloning, polymorphism and organization expression of AA‐NAT, ASMT and TAC3 gene in small tail han sheep. Master degree, Yangzhou University, China.

[vms3301-bib-0058] Tan, I. A. , Frewin, K. , Ricciardelli, C. , & Russell, D. L. (2019). ADAMTS1 promotes adhesion to extracellular matrix proteins and predicts prognosis in early stage breast cancer patients. Cellular Physiology and Biochemistry, 52, 1553–1568. 31135123 10.33594/000000108

[vms3301-bib-0059] Tola, E. N. , Karatopuk, D. U. , Koroglu, N. , Ergin, M. , & Oral, H. B. (2017). Follicular ADAMTS‐1 and aggrecan levels in polycystic ovary syndrome. Journal of Assisted Reproduction and Genetics, 34, 811–816. 10.1007/s10815-017-0913-7 28417352 PMC5445050

[vms3301-bib-0060] Tu, Y. R. (1989). The sheep and goat breeds in China. Shanghai, China: Shanghai Science and Technology Press.

[vms3301-bib-0061] Wang, L. , Li, J. , Ruan, Y. , Lu, T. , Liu, C. , Jia, M. , … Zhang, D. (2013). Sequencing ASMT identifies rare mutations in Chinese Han patients with autism. PLoS One, 8, e53727 10.1371/journal.pone.0053727 23349736 PMC3547942

[vms3301-bib-0062] Willis, E. L. , Bridges, P. J. , & Fortune, J. E. (2017). Progesterone receptor and prostaglandins mediate luteinizing hormone‐induced changes in messenger RNAs for ADAMTS proteases in theca cells of bovine periovulatory follicles. Molecular Reproduction and Development, 84, 55–66. 10.1002/mrd.22761 27879029 PMC5316468

[vms3301-bib-0063] Wittes, J. , & Schupbach, T. (2019). A gene expression screen in drosophila melanogaster identifies novel JAK/STAT and EGFR targets during oogenesis. G3: Genes, Genomes, Genetics, 9, 47–60. 30385460 10.1534/g3.118.200786 PMC6325903

[vms3301-bib-0064] Yuan, W. , Matthews, R. T. , Sandy, J. D. , & Gottschall, P. E. (2002). Association between protease‐specific proteolytic cleavage of brevican and synaptic loss in the dentate gyrus of kainate‐treated rats. Neuroscience, 114, 1091–1101. 10.1016/S0306-4522(02)00347-0 12379262

[vms3301-bib-0065] Yue, F. , Cheng, Y. , Breschi, A. , Vierstra, J. , Wu, W. , Ryba, T. , … Ren, B. (2014). A comparative encyclopedia of DNA elements in the mouse genome. Nature, 515, 355–364. 10.1038/nature13992 25409824 PMC4266106

